# Mito-TEMPO mitigates 5-fluorouracil-induced intestinal injury via attenuating mitochondrial oxidative stress, inflammation, and apoptosis: an in vivo study

**DOI:** 10.1007/s10787-023-01261-6

**Published:** 2023-06-20

**Authors:** Prasad Kisan Tambe, H. S. Qsee, Sanjay Bharati

**Affiliations:** Department of Nuclear Medicine, Manipal College of Health Professions, Manipal Academy of Higher Education, Manipal, Karnataka India

**Keywords:** Intestinal toxicity, Chemotherapy, Mitochondrial oxidative stress, Mitochondrial dysfunction, Mitochondria-targeted antioxidant, Combinatorial therapy

## Abstract

**Background:**

Recent evidences highlight role of mitochondria in the development of 5-fluorouracil (5-FU)-induced intestinal toxicity. Mitochondria-targeted antioxidants are well-known for their protective effects in mitochondrial oxidative stress- mediated diseases. In the present study, we investigated protective effect of Mito-TEMPO in 5-FU-induced intestinal toxicity.

**Methods:**

Mito-TEMPO (0.1 mg/kg b.w.) was administered intraperitoneally to male BALB/c mice for 7 days, followed by co-administration of 5-FU for next 4 days (intraperitoneal 12 mg/kg b.w.). Protective effect of Mito-TEMPO on intestinal toxicity was assessed in terms of histopathological alterations, modulation in inflammatory markers, apoptotic cell death, expression of 8-OhDG, mitochondrial functional status and oxidative stress.

**Results:**

5-FU administered animals showed altered intestinal histoarchitecture wherein a shortening and atrophy of the villi was observed. The crypts were disorganized and inflammatory cell infiltration was noted. Mito-TEMPO pre-protected animals demonstrated improved histoarchitecture with normalization of villus height, better organized crypts and reduced inflammatory cell infiltration. The inflammatory markers and myeloperoxidase activity were normalized in mito-TEMPO protected group. A significant reduction in intestinal apoptotic cell death and expression of 8-OhDG was also observed in mito-TEMPO group as compared to 5-FU group. Further, mtROS, mtLPO and mitochondrial antioxidant defense status were improved by mito-TEMPO.

**Conclusion:**

Mito-TEMPO exerted significant protective effect against 5-FU-induced intestinal toxicity. Therefore, it may be used as an adjuvant in 5-FU chemotherapy.

## Introduction

5-Fluorouracil (5-FU) is an antimetabolite drug which is frequently used to treat cancer, especially colorectal cancer (CRC). 5-FU exhibits its anticancer activity by inhibiting thymidylate synthase enzyme and incorporating its toxic metabolites into RNA and DNA which ultimately leads to cell death (Atiq et al. 2019). Since 5-FU acts not only on cancer or tumor cells, but also on normal proliferative cells, therefore, gastrointestinal toxicity is one of the most prevalent adverse effects reported during systemic CRC therapy (Al-Asmari et al. 2015; Lee et al. 2014). Although, the molecular mechanism of 5-FU-induced toxicities is still not entirely understood, oxidative stress (O.S.) caused by reactive oxygen species and mitochondrial dysfunction are frequently mentioned in in-vivo and in-vitro research (Diba et al. 2021; Ishibashi et al. 2021; Rapa et al. 2021). A recent study on human intestinal organoids confirmed the downregulation of several genes responsible for ATP synthesis, assembly of mitochondrial complex I proteins and mitochondrial uncoupling proteins during 5-FU administration leading to overall mitochondrial dysfunction (Rodrigues et al. 2021). Another in vivo investigation reported ultrastructural abnormalities in mitochondria, such as swollen and degenerated mitochondria with disintegrating cristae, after 5-FU exposure to intestinal cells (Koli et al. 2014). The generation of ROS by NADPH oxidase (NOX) has been linked to TNF-α induced apoptosis in the rat intestinal epithelial cells-6 (IEC-6), whereas mitochondrial dysfunction along with increased mitochondrial ROS generation, has been proposed as a cause of TNF-α induced apoptosis in the rat IEC (cell line RIE-1) (Babu et al. 2015).

Regardless of the role of oxidative stress in 5-FU-mediated toxicity, traditional antioxidants failed to attenuate the adverse effects of this anticancer drug (Ambroz et al. 2019; Fujimoto et al. 1983; Legha et al. 1982). One of the factors responsible for this might be non-specific nature of these antioxidants and thus, lack of availability at the location of ROS generation i.e., mitochondria. Therefore, most efficient way to overcome this problem is to target antioxidants to the site of ROS generation. This can be effectively attained by the mitochondria-targeted antioxidants (Sacks et al. 2021; Toyama et al. 2018; Rocha et al. 2016).

Mitochondria-targeted antioxidants (MTAs) are combination of biologically active antioxidant which is conjugated with a carrier such as lipophilic cations, liposomes or peptides that helps bioactive antioxidant constituent to be transported to the mitochondria (Jiang et al. 2020). One of the MTAs, Mito-TEMPO, is a combination of antioxidant piperidine nitroxide (TEMPO) and lipophilic cation triphenylphosphonium (TPP^+^) where TPP^+^ helps in mitochondrial localization of antioxidant piperidine nitroxide. In our earlier studies, we have observed its effective anticancer activity in hepatocellular carcinoma animal model (Shetty et al. 2019). Since mito-TEMPO possess anticancer activity as well as targeted antioxidant activity therefore, this antioxidant may prove to be a good adjuvant in 5-FU chemotherapy, where toxicity is a serious concern. Therefore, in the present study, we explored modulatory effect of mito-TEMPO in 5-FU induced intestinal toxicity.

## Materials and methods

### Chemicals and reagents

5-Fluorouracil, Mito-TEMPO, DCFH-DA, and Rhodamine 123 were procured from Sigma–Aldrich, USA. Antibodies for detection of inflammatory markers such as IL-6 (bs-0782R), IL-10 (PA5-85,660), TNF-α (bs-2081R), and antibody for the detection of oxidative DNA damage marker i.e. 8-OHdG (bs-1278R) along with goat anti-rabbit IgG secondary antibody (65–6120) were procured from Thermo Fisher Scientific (Rockford, USA). R & D systems, USA provided TACS-XL In Situ Apoptosis Detection Kit. All other chemicals and reagents used in the present study were of highest purity grade and procured from local Indian firms.

### Animals and experimental treatment

**Fig. 1:**
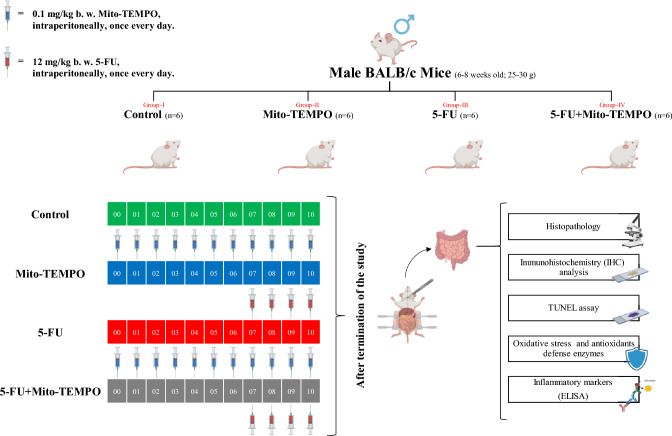
Schematic representation of animal grouping, dose regimen and experimental procedures

Animal experiments carried out in the present research were approved by Institutional animal ethics committee (IAEC/KMC/54/2021) and animals were handled in accordance with CPCSEA (Committee for the Purpose of Control and Supervision of Experiments on Animals) guidelines, Govt. of India. Male BALB/c mice in weight range of 25–30 g and 6–8 weeks old were housed under environmental conditions (room temperature 25 ± 1 °C; humidity (65–80%); 12/12 h alternate light/dark cycle). After 1 week of acclimatization period, animals were segregated into four groups (*n* = 6 in each group) viz, Control, Mito-TEMPO, 5-FU and 5-FU + Mito-TEMPO. Animals from 5-FU group received daily dose of 5-FU (12 mg/kg b.w.) intraperitoneally for four successive days. Mito-TEMPO group animals were administered with 0.1 mg/kg b.w. mito-TEMPO where, Mito-TEMPO treatment was initiated 1 week prior to the 5-FU treatment and continued till the termination of experiment. 5-FU + Mito-TEMPO treatment group received 5-FU and Mito-TEMPO doses in similar fashion as described for 5-FU and Mito-TEMPO groups earlier. Animals were sacrificed by cervical dislocation under mild anesthesia at the end of the study and small intestine tissue samples were processed for further toxicity study (Fig. 1).

### Diarrhea assessment

Upon initiation of dosing, animals from the different experimental groups were assessed for presence of diarrhea daily. Severity of the diarrhea was graded as no diarrhea (0 score), mild diarrhea (1 score), moderate diarrhea (2 score), Severe diarrhea (3 score) (Bowen et al. 2007).

### Histopathological analysis of duodenal tissue

The duodenal tissue sections from mice were stained using standard hematoxylin and eosin (H&E) staining method (Boeing et al. 2021). The H & E stained duodenal tissues were observed for histological alterations using light microscope (Lx 300, Labomed, USA).

### Assessment of myeloperoxidase (MPO) activity

The presence of the enzyme myeloperoxidase (MPO) in the intestine was shown to be a sign of acute inflammation and neutrophil infiltration. Briefly, pellets obtained after centrifugation of tissue homogenate was re-suspended in hexadecyl trimethylammonium bromide buffer. This suspension was sonicated on ice and re-centrifuged (10,000 × g; 15 min). Resultant supernatant was mixed with potassium phosphate buffer containing substrates as 0.167 mg/ml of O-dianisidine dihydrochloride and 20 mM H_2_O_2_. Change in optical density was noted at 450 nm for 3 min. Enzyme activity was expressed as units/min/mg protein (Bradley et al. 1982).

### Assessment of inflammatory markers

5-FU chemotherapy reportedly demonstrated the mucosal inflammation in intestine. The ELISA technique was used to measure the levels of IL-6, IL-10, and TNF-α in intestinal tissue. In brief, antigen extraction was carried out from mouse intestine tissue as described earlier (Shetty et al. 2019). 50 μL of extracted antigen was incubated overnight at 4 °C in 96 well polystyrene flat-bottom ELISA plates (Himedia, India). The plates were washed with wash buffer (PBSTw) before blocking with 1% bovine serum albumin. Additionally, 100 μL of primary IL-6, IL-10, and TNF- antibodies (1:1000 dilution) were added to the respective wells and incubated for 2 h at room temperature. Following the incubation period, ELISA plates were rinsed with wash buffer (PBSTw), 100 μL of secondary antibody (goat anti-rabbit IgG secondary antibody) was added to respective wells, and the plates were left to incubate at room temperature (1 h). Finally, 3,3′,5,5′-tetramethylbenzidine substrate, a coloring agent, was added to each well. H_2_SO_4_ (2 M) was used to terminate the reaction, and an ELISA plate reader (BioTek, SYNERGY H1) was used to measure optical density at 450 nm.

### Cell death analysis

Immunohistostaining of intestinal tissues were carried out to assess the cell death by TUNEL assay technique (TACS-XL In Situ Apoptosis Detection Kit, R & D systems, USA). Briefly, tissue sections were deparaffinized, rehydrated, and incubated with proteinase K (37 °C for 15 min). Slides were washed with deionizing water for 2 min and quenched for 5 min in quenching solution (30% H_2_O_2_ in methanol). Further, slides were washed with PBS, immersed in 1X TdT labeling buffer, and incubated with B-dNTP labelling reaction mixture in a humidifying chamber (37 °C for 30 min). Furthermore, slides were transferred to stop buffer solution (at room temperature for 5 min), washed with PBS, and incubated with Anti-BrdU antibody solution in humidifying chamber (37 °C for 30 min). After washing with PBS-Tween 20, slides were immersed in Strep-HRP solution and counterstained with methyl green. The stained slides were observed under light microscope (Lx 300, Labomed, USA).

### Assessment of chemotherapy-induced oxidative DNA damage

Immunohistochemical analysis for 8-OHdG was carried out in intestinal tissue as described earlier (Qsee et al. 2022). In brief, intestinal tissue Sects. (5 μm) were dewaxed, rehydrated in alcohol, and submerged in 10 mM sodium citrate solution (pH 6) for epitope retrieval. The endogenous peroxidase activity and non-specific binding sites were blocked using 1% H_2_O_2_ and 10% BSA respectively. Further, slides were incubated in primary antibody 8-OHdG, rinsed with PBS, and again incubated in HRP-labeled-secondary antibody. The slides were then treated with 1% 3, 3'-diaminobenzidine and mounted for microscopic examinations.

### Mitochondrial isolation

Intestinal mitochondria were isolated as described earlier (Sun et al. 2010). In brief, intestinal tissues were homogenized in ice cold homogenizing buffer (0.25 M sucrose, 10 mM Tris–HCl, 0.5 mM EDTA; pH 7.4) using homogenizer (Remi RQT-127AD, India). Obtained homogenate was centrifuged at 2000 × g (15 min), supernatant was collected, and re-centrifuged at 14,000 × g (20 min). Finally, pellet was resuspended in suspension buffer (10 mM Tris–HCL, 0.25 M sucrose; pH 7.8) for further use.

### Assessment of mitochondrial oxidative stress

Mitochondrial reactive oxygen species (mtROS) and mitochondrial lipid peroxidation (mtLPO) were used as indicators of mitochondrial oxidative stress. mtROS and mtLPO were estimated using standard laboratory protocols as described earlier (Keshtzar et al. 2016; Tabassum et al. 2010). For estimation of mtROS, optical density of the solution was noted in microplate reader (BioTek, SYNERGY H1) at wavelength of 500 nm (excitation) and 520 nm (emission).

### Estimation of mitochondrial complexes enzyme and TCA cycle enzymes activities

Mitochondrial complex-I, complex-II, and complex-IV enzyme activities were estimated as described by (Minakami et al. 1962; Pearl et al. 1962). Further, activities of mitochondrial TCA cycle enzymes such as isocitrate dehydrogenase (IDH) and malate dehydrogenase (MDH) were estimated as described by (Beshbishy et al. 2013; Macnicol et al. 1992).

### Estimation of mitochondrial membrane potential (MMP)

Mitochondrial membrane potential was estimated according to the method described earlier (Bhardwaj et al. 2018). In brief, mitochondrial suspension was added to the reaction mixture (150 mM sucrose, 4 mM MgCl_2_, 30 mM HEPES KOH, 5 mM K_2_HPO_4_), incubated (37 °C, 5 min), and added with 5 μM Rhodamine 123 to initiate reaction. Optical density of the solution was noted in microplate reader (BioTek, SYNERGY H1) at wavelength of 507 nm (excitation) and 527 nm (emission).

### Mitochondrial antioxidant defence system status

Mitochondrial antioxidant defence status of mice intestine was estimated in terms of activities of mitochondrial reduced glutathione (mtGSH), mitochondrial glutathione reductase (mtGR), mitochondrial glutathione peroxidase (mtGPx) and mitochondrial manganese superoxide dismutase (MnSOD). Activity of mtGSH was estimated according to (Ellman 1959). The activity of mtGR was estimated as described by (Mcfarland et al. 1999). In addition, the activity of mtGPx was estimated according to (El-Beshbishy et al. 2013). Furthermore, activity of MnSOD was estimated as described by (Bindhumol et al. 2003). Absorbance was measured spectrophotometrically using multiplate reader (BioTek, SYNERGY H1).

### Protein estimation

Protein concentration in intestinal mitochondrial fraction was estimated using Lowrey’s method (Lowery et al. 1951). Bovine serum albumin (BSA) was used as protein standard. O.D. of the sample was noted at 620 nm spectrophotometrically using multiplate reader (BioTek, SYNERGY H1).

### Statistical analysis

Normality of the data was checked using Shapiro–Wilk test and homogeneity of variance was estimated using Leven’s test. The intergroup comparison of different parameters was estimated using one-way ANOVA followed by Tukey’s HSD (post hock) test. Statistical significance was kept at *P* ≤ 0.05.

## Results

### Mito-TEMPO pre-treatment preserved the overall histoarchitecture, mitigated the oxidative DNA damage, apoptosis, and inflammation in intestinal tissue

**Fig. 2:**
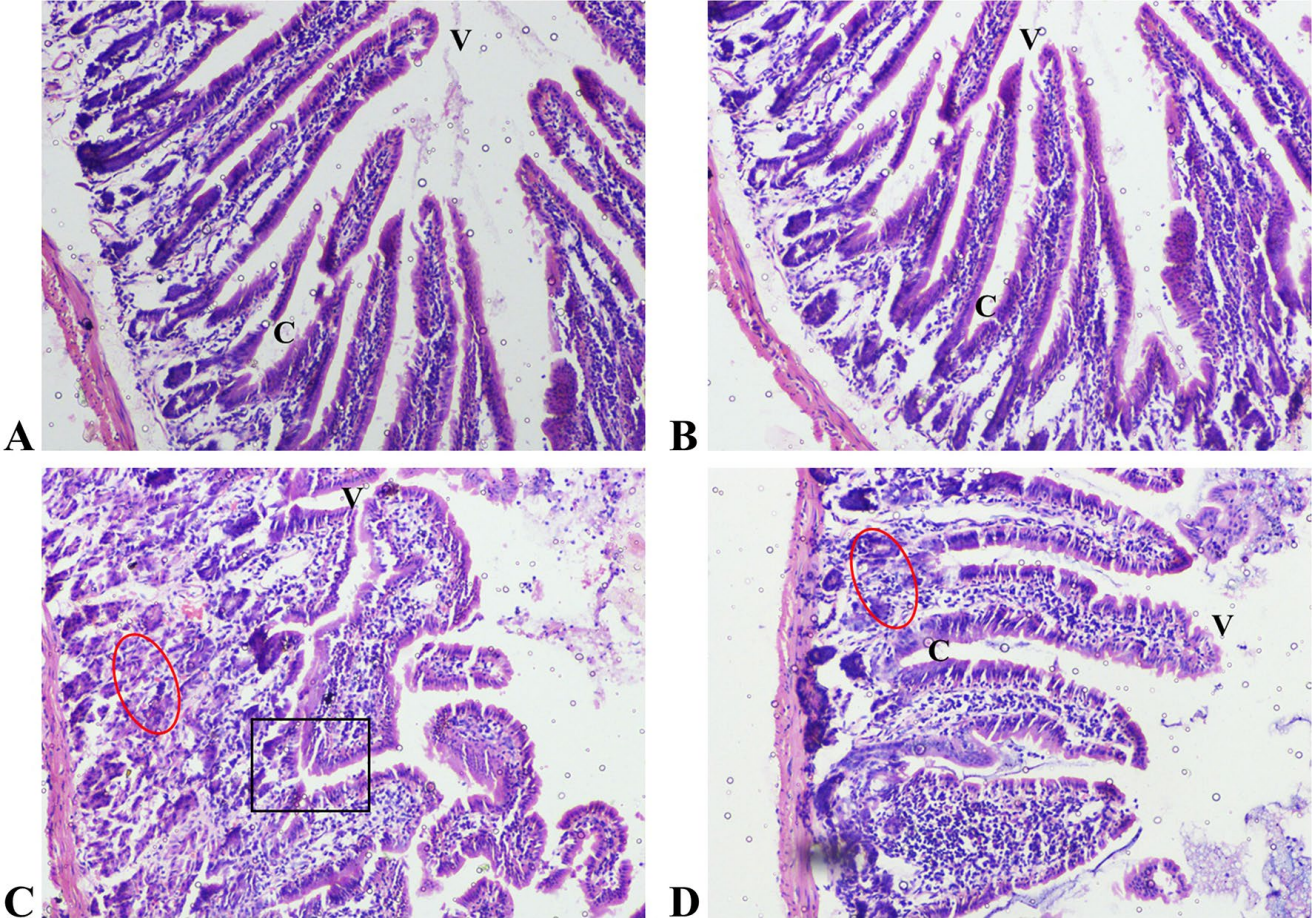
Histoarchitecture of duodenal tissue in different treatment groups **A** Control group and **B** Mito-TEMPO group animals demonstrated normal villus height and crypts architecture without any inflammatory cell infiltration. **C** 5-FU challenged group animals demonstrated decreased villus height (v), crypt disorganization (black square) and intense inflammatory cell infiltration (circle). **D** 5-FU + Mito-TEMPO group animals demonstrated reversal of altered villus height (v), conserved crypts histoarchitecture (**c**) and reduced inflammatory cell infiltration (circle) (Magnification 400x; Scale bar = 200 μm)

Four-day treatment of 5-FU at dose of 12 mg/ kg b.w. significantly altered the overall histoarchitecture of mice intestine. H and E-stained intestinal tissue sections obtained from 5-FU challenged animals demonstrated intense inflammatory cell infiltration (red circle); shortening, atrophy of the villi (indicated by “V”) and marked disorganization of crypts (black square) **(**Fig. 2C**)**. Pre-treatment with mito-TEMPO suppressed 5-FU induced histological damage and improved the overall histoarchitecture in terms of normal villus height, decreased crypts disorganization and inflammatory cells infiltration **(**Fig. 2D**).**

**Fig. 3:**
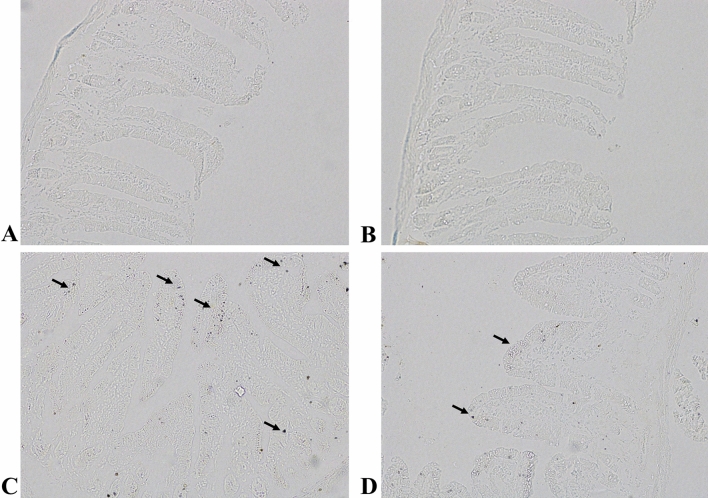
Expression of 8-OHdG in different treatment groups **A** Control group; demonstrates uniformly stained, light intestinal cells **B** Mito-TEMPO group; demonstrates similar staining pattern as compared to control group. **C** 5-FU group; demonstrates localization of 8-OHdG within enterocytes (dark brown spots indicated by arrow) **D** 5-FU + Mito-TEMPO group; demonstrates decreased number of dark brown spots (arrow) as compared to 5-FU group. (Magnification 400x; Scale bar = 200 μm)

Further, oxidative DNA damage in different treatment group was demonstrated by 8-OHdG immunostaining. Control and Mito-TEMPO group animals demonstrated normal, light, and uniformly stained intestinal cells **(**Fig. 3A and B**)**. 5-FU group animals showed increased intensity of 8-OHdG immunostaining demonstrated by dark brown spots indicating 8-OHdG positive enterocytes (arrowhead) **(**Fig. 3C**)**. However, pre-treatment with mito-TEMPO demonstrated lower number of positively stained enterocytes as compared to 5-FU group **(**Fig. 3D**)**.

**Fig. 4:**
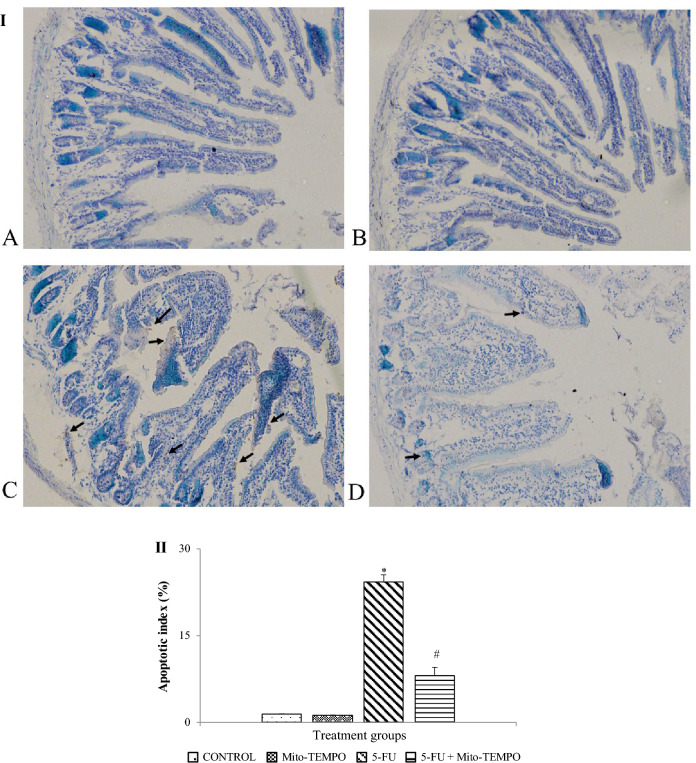
**I** 5-FU-induced apoptotic cell death in different treatment groups **A** Control group; demonstrates normal intestinal cells with no visible apoptotic cells **B** Mito-TEMPO group; showing normal intestinal cells with absence of any apoptotic cells **C** 5-FU group; showing darkly stained brown color TUNEL positive cells (black arrow) indicative of increased apoptosis **D** 5-FU + Mito-TEMPO group; showing decreased expression of TUNEL positive cells **II **Percent apoptotic index; the graph shows significant increase in percent apoptotic index in 5-FU group as compared to Control group. Significant decrease in percentage of apoptotic cells was observed in 5-FU + Mito-TEMPO group as compared to 5-FU group. (Data were presented mean ± SD. Data analysis was carried out using one-way ANOVA followed by post hoc test (Tukey’s HSD). *p ≤ 0.05 when compared to Control. #: represents *p* ≤ 0.05 when compared to 5-FU.)

Further, TUNEL stained sections of mice intestine from different treatment groups were observed for cell death analysis. Control and Mito-TEMPO group demonstrated normal enterocytes with negligible TUNEL positive cells. The apoptotic index of Control and Mito-TEMPO groups were 1.4 ± 0.0625 and 1.17 ± 0.0450 respectively (Fig. 4IA and IIB). The 5-FU and 5-FU + Mito-TEMPO groups showed brown color TUNEL positive cells (black arrow) (Fig. 4IC and ID). The apoptotic index was significantly (p ≤ 0.05) increased in 5-FU group (24.23 ± 2.0362). However, pre-treatment with mito-TEMPO significantly (p ≤ 0.05) decreased the apoptotic index (8.09 ± 1.4631) when compared to 5-FU group.

Further, to study the effect of Mito-TEMPO on pro-inflammatory markers in 5-FU-induced intestinal injury we performed ELISA and results were shown in **(**Table 1**)**. A significantly (*p* ≤ 0.05) higher expression of IL-6 and TNF-α were observed in animals administered with 5-FU when compared to Control group animals. Mito-TEMPO treatment to 5-FU group animals in 5-FU + Mito-TEMPO group demonstrated significant (*p* ≤ 0.05) decrease in the expression of IL-6 (22.06%) and TNF-α (43.68%) when compared to 5-FU group. Further, a significant (*p* ≤ 0.05) drop in the expression of IL-10 was observed in 5-FU group when compared to Control group. However, Mito-TEMPO treatment significantly (*p* ≤ 0.05) increased the expression of IL-10 in 5-FU + Mito-TEMPO group when compared to 5-FU group. In addition, we also determined the myeloperoxidase (MPO) activity which is a biochemical marker of the infiltration of granulocytes and important parameter for detection of intestinal inflammation during oxidative stress. Interestingly, MPO demonstrated significant (*p* ≤ 0.05) decrease in its activity i.e., 14.71% in 5-FU + Mito-TEMPO group as compared to 5-FU group.

**Table 1:** Effect of mito-TEMPO on inflammatory markers in intestinal tissue

Parameters	Control	Mito-TEMPO	5-FU	5-FU + Mito-TEMPO
IL-6 proteins (OD/mg tissue protein)	5.52 ± 0.69	5.68 ± 0.47	9.11 ± 0.53*	7.10 ± 0.46*^£#^
IL-10 proteins (OD/mg tissue protein)	6.14 ± 0.30	6.39 ± 0.30	3.29 ± 0.48*	4.94 ± 0.11*^£#^
TNF- α proteins (OD/mg tissue protein)	5.92 ± 0.17	5.50 ± 0.40	10.92 ± 0.49*	6.15 ± 0.30*^£#^
MPO (U/mg tissue protein)	25.68 ± 1.31	26.28 ± 0.59	35.54 ± 0.81*	30.31 ± 0.70*^£#^

Data were presented as mean ± SD. Data analysis was carried out using one-way ANOVA with subsequent application of post hoc test (Tukey’s HSD). *p ≤ 0.05 when compared to Control. £: *p* ≤ 0.05 when compared to mito-TEMPO. #*p* ≤ 0.05 when compared to 5-FU

### Mito-TEMPO mitigated the 5-FU induced mitochondrial oxidative stress and improved mitochondrial antioxidant defence status of intestinal tissue

The levels of mtLPO and mtROS were evaluated in the intestinal tissue to determine the mitochondrial oxidative stress. mitoLPO and mtROS levels were significantly (*P* ≤ 0.05) increased after 5-FU treatment to animals in 5-FU group when compared to Control group. However, mito-TEMPO treatment in 5-FU + Mito-TEMPO group demonstrated significantly decreased levels of mtLPO (17.74%) and mtROS (27.27%) when compared to 5-FU group (Table 2) which indicates role of mito-TEMPO in decreasing mitochondrial oxidative stress in 5-FU-induced intestinal injury.

**Table 2:** Mitochondrial oxidative stress and mitochondrial antioxidant defense system in different treatment groups

Parameters	Control	Mito-TEMPO	5-FU	5-FU + Mito-TEMPO
Mitochondrial LPO (nmol/ min/ mg protein)	0.54 ± 0.03	0.51 ± 0.01	0.62 ± 0.008*	0.55 ± 0.04^#^
Mitochondrial ROS (AFU)	149.24 ± 5.47	141.02 ± 5.15	225.24 ± 7.43*	163.08 ± 8.59^#^
Mitochondrial GSH (nmol/mg mitochondrial protein)	0.74 ± 0.03	0.75 ± 0.04	0.58 ± 0.01*	0.68 ± 0.01*^£ #^
Mitochondrial GR (nmol/min/mg Mitochondrial protein)	0.38 ± 0.007	0.37 ± 0.021	0.15 ± 0.008*	0.28 ± 0.012*^£ #^
Mitochondrial GPx (nmole/min/mg Mitochondrial protein)	1.25 ± 0.03	1.25 ± 0.05	0.50 ± 0.04*	0.86 ± 0.02*^£ #^
Mitochondrial SOD (IU/mg mitochondrial protein)	0.39 ± 0.01	0.37 ± 0.02	0.22 ± 0.01*	0.30 ± 0.01*^£ #^

Data were expressed as mean ± SD and analyzed using one-way ANOVA followed by post hoc test (Tukey’s HSD). *represents *p* ≤ 0.05 when compared with the Control group. £: represents *p* ≤ 0.05 when compared with mito-TEMPO group. #represents *p* ≤ 0.05 when compared with the 5-FU group

In order to highlight the involvement of O.S. in 5-FU-induced intestinal injury, we further assessed the activities of mitochondrial antioxidant defence enzymes, which is often helpful in maintaining redox homeostasis. Mitochondrial antioxidant defence system was also derailed in 5-FU group. Significant (*P* ≤ 0.05) decrease in activities of mtGSH, mtGPx, mtGR and MnSOD were observed in 5-FU group as compared to Control group **(**Table 2**)**. However, mito-TEMPO treatment significantly (*P* ≤ 0.05) increased the activities of mtGSH (17.24%), mtGPx (72%), mtGR (86.66%) and MnSOD (36.36%) in 5-FU + Mito-TEMPO group as compared to 5-FU group **(**Table 2**)**. These findings comprehensively highlight protective role of mito-TEMPO in 5-FU-induced intestinal injury.

### Mito-TEMPO modulated the mitochondrial functioning and restored mitochondrial membrane potential in 5-FU-induced intestinal injury

Since the mitochondrial oxidative stress may have resulted from defective mitochondrial functioning, we were further encouraged to assess activities of respiratory chain enzymes, TCA cycle enzymes and mitochondrial membrane potential in mice intestine. 5-FU challenged animals demonstrated significant (*P* ≤ 0.05) reduction in the enzymatic activities of mitochondrial complex I, mitochondrial complex II, and mitochondrial complex IV when compared to Control group. Mito-TEMPO treatment significantly increased the activities of mitochondrial complex I (53.15%), complex II (44.98%), and complex IV (57.14%) when compared to 5-FU group **(**Table 3**)**. Further, the activities of IDH and MDH were significantly (*P* ≤ 0.05) reduced in 5-FU exposed animals when compared to Control group. However, mito-TEMPO pre-treatment significantly (*P* ≤ 0.05) increased the activities of IDH (45.11%) and MDH (40.71%) **(**Table 3**)**. The mitochondrial damage were further demonstrated in 5-FU group by significant (*P* ≤ 0.05) drop in mitochondrial membrane potential as compared to Control group. However, mito-TEMPO treatment significantly increased the mitochondrial membrane potential (49.15%) in 5-FU + mito-TEMPO group as compared to 5-FU group **(**Table 3**)**.

**Table 3:** Effect of Mito-TEMPO on activities of mitochondrial enzymes and mitochondrial membrane potential in different treatment groups

Parameters	Control	Mito-TEMPO	5-FU	5-FU + Mito-TEMPO
Complex I (nmole NADH oxidized/min/mg mitochondrial protein)	2.10 ± 0.11	2.09 ± 0.04	1.11 ± 0.12*	1.70 ± 0.05*^£ #^
Complex II (nmol/min/mg mitochondrial protein)	104.85 ± 6.37	103.14 ± 3.78	58.22 ± 2.81 *	84.41 ± 5.46*^£ #^
Complex IV (nmole NADH oxidized/min/mg mitochondrial protein)	0.74 ± 0.01	0.74 ± 0.02	0.35 ± 0.008*	0.55 ± 0.013*^£ #^
MDH (nmol NADH oxidized/min/mg mitochondrial protein)	107.08 ± 3.66	106.36 ± 4.39	59.44 ± 3.10*	83.64 ± 3.92*^£ #^
IDH (nmole NADH oxidized/min/mg mitochondrial protein)	2.14 ± 0.13	2.16 ± 0.13	1.33 ± 0.06*	1.93 ± 0.04*^£ #^
MMP Relative Intensity (AFU)	518.02 ± 16.94	506.84 ± 19.06	278.12 ± 10.30*	414.84 ± 12.23*^£ #^

Data were expressed as mean ± SD and analyzed using one-way ANOVA followed by post hoc test (Tukey’s HSD). *represents *p* ≤ 0.05 when compared with the Control group. £represents *p* ≤ 0.05 when compared with mito-TEMPO group. #represents *p* ≤ 0.05 when compared with the 5-FU group

## Discussion

Anti-neoplastic drug-induced gastrointestinal toxicity is a multistep process that may include direct DNA damage to normally proliferating cells, an increase in ROS production and activation of several transcription factors (Chamseddine et al. 2019; Al-Asmari et al. 2015). 5-Fluorouracil is a well-known anti-neoplastic drug which shows several undesirable effects on gastrointestinal system (Chen et al. 2022; Gui et al. 2022). After administration of 5-FU, fluorodeoxyuridine monophosphate (F-dUMP) is formed as a primary metabolite which inhibits the activity of thymidylate synthase enzyme. This enzyme plays a major role in synthesis of thymidine monophosphate (dTMP) from deoxyuridine monophosphate (dUMP). The depletion in dTMP levels in the cells eventually decrease dTTP levels which are important for DNA synthesis in fast proliferating cells. The inhibition of thymidylate synthase also increases the levels of deoxyuridine monophosphate (dUMP) which is then converted to dUTP and get misincorporated into DNA instead of dTTP. These ineffective cycles of misincorporation, excision, and repair eventually results in DNA strand breakage and cell death. (Raffa and Tallarida 2011; Longley et al. 2003). This mechanism is thought to be responsible not only for reduction of cancer cell growth but also the normal tissues such as intestinal epithelial cells (Wei et al. 2020). Additionally, recent evidences suggest that mitochondria also play a significant role in the development of 5-FU intestinal toxicity (Yim et al. 2021; Rodrigues et al. 2021; Hudita et al. 2021). The ATP synthesis is significantly affected in case of 5-FU-induced intestinal toxicity (Jardi et al. 2022). According to Rodrigues et al. 2021 a decrease in ATP production is associated with lower expression of genes such as ACAD9 and UCP2, that are essential in assembling mitochondrial complex I proteins and mitochondrial uncoupling proteins. Since these proteins are crucial components of mitochondrial functioning and play a vital role in cellular respiration, decreased expression of these genes indicates an altered ATP synthesis via the mitochondrial respiratory electron transport chain (Rodrigues et al. 2021). Another in vivo study also demonstrated ultra-structural defects in mitochondria of intestinal cells such as swollen and degenerated mitochondria with disintegrated cristae after 5-FU administration (Gawish et al. 2013). Considering this in the present study we attempted to understand how modulation of mitochondrial oxidative stress by mitochondria-targeted antioxidant, Mito-TEMPO affects 5-FU-induced intestinal injury.

After administration of 5-FU to the animals, the histological abnormalities in the intestinal tissue were clearly visible. A substantial number of inflammatory cells, atrophy of villi and marked disorganization of crypts were observed in the intestinal tissue. These changes indicated towards chemotherapy-induced mucositis. During initiation phase of mucositis, a lot of free radicals are generated which results in oxidative stress. Several studies have demonstrated the involvement of mitochondria in chemotherapy-induced mucositis (Avila et al. 2022; Hu et al. 2018; Natarajan et al. 2017, 2016; Koli et al. 2014). However, the protective effect of Mito-TEMPO in 5-FU toxicity was clearly observed in histopathology of intestinal tissue. Mito-TEMPO protected group improved the overall histoarchitecture indicating protective potential of mito-TEMPO.

Yanez et al. 2003 demonstrated involvement of mitochondrial DNA in cisplatin and 5-fluorouracil-induced gastrointestinal damage in rats. Further, the author demonstrated that this damage was restricted to mitochondrial DNA instead of nuclear DNA, which might be because of limited mitochondrial repair pathways (Yanez et al. 2003). The rise in the mitochondrial ROS and increased 8-OHdG expression upon 5-FU administration to the animals in the present study might indicated the similar effect. 8-hydroxy-2-deoxyguanosine (8-OHdG) is one of the most common forms of oxidative lesions caused by free radicals in nuclear and mitochondrial DNA. It is often referred as biomarker for oxidative stress (Valavanidis et al. 2009; Xu et al. 2008). However, pre-treatment of mito-TEMPO to 5-FU challenged animals significantly decreased the expression of this marker along with mitochondrial ROS highlighting the importance of targeted antioxidant therapy.

Further, the impairment of mitochondrial intrinsic antioxidant defence pathways contributed to deteriorating effects of 5-FU. In mitochondria, two mechanisms work together to reduce oxidative damage; the superoxide radical dismutation system and the peroxides elimination system. (Dutordoir and Bates 2021; Bharati and Shetty 2020). These two antioxidant defence mechanisms work together to remove oxidative damage. Superoxide dismutase system acts on the superoxide free radicals and convert highly reactive superoxide free radicals to less reactive hydrogen peroxide. The comparatively less toxic hydrogen peroxide can also be potentially detrimental if it undergoes the Fenton reaction, which produces hydroxyl free radicals (Stanbury 2022). Therefore, second line of mitochondrial antioxidant defense system which consists of thioredoxin-dependent enzyme peroxiredoxin III, glutathione peroxidase, and glutathione reductase play a potent antioxidant role in degradation of mitochondrial hydrogen peroxide/peroxides. After 5-FU administration these systems were derailed in the intestinal tissue and a significant decrease in the activity was noted. Our findings were in concordance with other studies where a similar depression in the activity of mitochondrial enzymes was reported by researchers after 5-FU administration (Xia et al. 2022; Santandreu et al. 2011). However, mito-TEMPO protected group showed significant improvement in the status of mitochondrial antioxidant defense system indicating its protective effect.

Another detrimental effect of oxidative stress in 5-FU-induced injury in intestinal tissue is mainly observed as upregulation of nuclear factor kappa-B (NF-κB) and other inflammatory mediators (Stringer et al. 2013). NF-κB promotes gene expression and the generation of pro-inflammatory cytokines such as tumor necrosis factor (TNF- α), interleukin (IL-1 β), and IL-6, which results in tissue damage and apoptosis (Liu et al. 2017). To confirm the up-regulation of inflammatory mediators, we estimated the levels of inflammatory cytokines such as IL-6, IL-10 and TNF-α by ELISA method. Additionally, we also determined myeloperoxidase (MPO) activity which is the most abundant pro-inflammatory biomarker present in neutrophilic granulocytes (Chami et al. 2018). Increased levels of this biomarker in circulation are attributed to elevated oxidative stress and inflammation (Ndrepepa 2019). In the present study, the exposure of 5-FU resulted in a significant increase in levels of inflammatory markers. However, Mito-TEMPO treatment to 5-FU exposure group mitigated the activity of MPO as well as decreased levels of inflammatory markers IL-6 and TNF-α. The inflammatory cytokines can also trigger apoptotic cell death in the intestinal tissue which is mediated through TNF receptor (Zhao et al. 2022; Ji et al. 2022; Peltzer and Walczak 2019). Therefore, we employed TUNEL assay method to detect 5-FU-induced intestinal cell death and observed that 5-FU treatment significantly increased apoptotic cell death. However, Mito-TEMPO treatment significantly decreased this apoptotic index which can be inferred as protective effect of Mito-TEMPO against 5-FU-induced injury.

## Conclusion

The results of the present study demonstrated that mitochondria-targeted antioxidant, Mito-TEMPO was effective in reducing 5-FU induced mitochondrial oxidative stress in intestinal tissue. This reduction in mitochondrial oxidative stress was subsequently translated into the overall protective effect on intestinal tissue. We have already observed anticancer activity of mitochondria-targeted antioxidants such as Mito-TEMPO and mitoQ in our previous studies therefore, in light of the present findings it may be suggested that mitochondria-targeted antioxidants can be a promising agent for combinatorial therapy.

## Data Availability

Data are available and can be provided upon a request to corresponding author.
